# Validation of a fast prognostic score for risk stratification of normotensive patients with acute pulmonary embolism

**DOI:** 10.1007/s00392-019-01593-w

**Published:** 2020-02-06

**Authors:** Lukas Hobohm, Cecilia Becattini, Stavros V. Konstantinides, Franco Casazza, Mareike Lankeit

**Affiliations:** 1 Center for Thrombosis and Hemostasis (CTH), University Medical Center of the Johannes Gutenberg University, Mainz, Germany; 2 Center for Cardiology, Cardiology I, University Medical Center of the Johannes Gutenberg-University, Mainz, Germany; 3 Internal and Cardiovascular Medicine-Stroke Unit, University of Perugia, Perugia, Italy; 4 Department of Cardiology, Democritus University of Thrace, Alexandroupolis, Greece; 5 Cardiology Department, San Carlo Borromeo Hospital, Milan, Italy; 6 Department of Internal Medicine and Cardiology, Campus Virchow Klinikum (CVK), Charité - University Medicine Berlin, Augustenburger Platz 1, 13353 Berlin, Germany

**Keywords:** Pulmonary embolism, Risk stratification, Algorithm, Guideline, Modified FAST score

## Abstract

**Background:**

Recent studies demonstrate an improved prognostic performance of the 2014 European Society of Cardiology (ESC) algorithm for risk stratification of patients with pulmonary embolism (PE) compared to the 2008 ESC algorithm. The modified FAST and Bova scores appear especially helpful to identify PE patients at intermediate-high risk.

**Methods:**

We validated the prognostic performance of the modified FAST score compared to other scores for risk stratification in a post-hoc analysis of 868 normotensive PE patients included in the prospective Italian Pulmonary Embolism Registry. In-hospital adverse outcome was defined as PE-related death, mechanical ventilation, cardiopulmonary resuscitation or administration of catecholamines.

**Results:**

Overall, 27 patients (3.1%) had an adverse outcome and 32 patients (3.7%) died. The rate of an adverse outcome was highest in the intermediate-high risk classes of the 2019 ESC algorithm (7.5%) and the modified FAST score (5.3%) while the Bova score failed to discriminate between intermediate-low and intermediate-high-risk patients. Patients classified as intermediate-high risk by the 2019 ESC algorithm (Odds Ratio [OR], 4.2 [95% CI, 1.9–9.0]) and modified FAST score (OR, 2.8 [1.3–6.2]) had a higher risk of an adverse outcome compared to patients classified by the Bova score (OR, 1.6 [0.7–3.7]). The c-index was higher for the 2019 ESC algorithm and the modified FAST score (AUC, 0.69 [0.58–0.79] and 0.67 [0.59–0.76]) compared to the Bova score (AUC, 0.64 [0.55–0.73]).

**Conclusions:**

The 2019 ESC algorithm provided the best prognostic performance, but also the modified FAST score accurately stratified normotensive PE patients in different risk classes while the Bova score failed to identify patients at highest risk.

**Graphic abstract:**

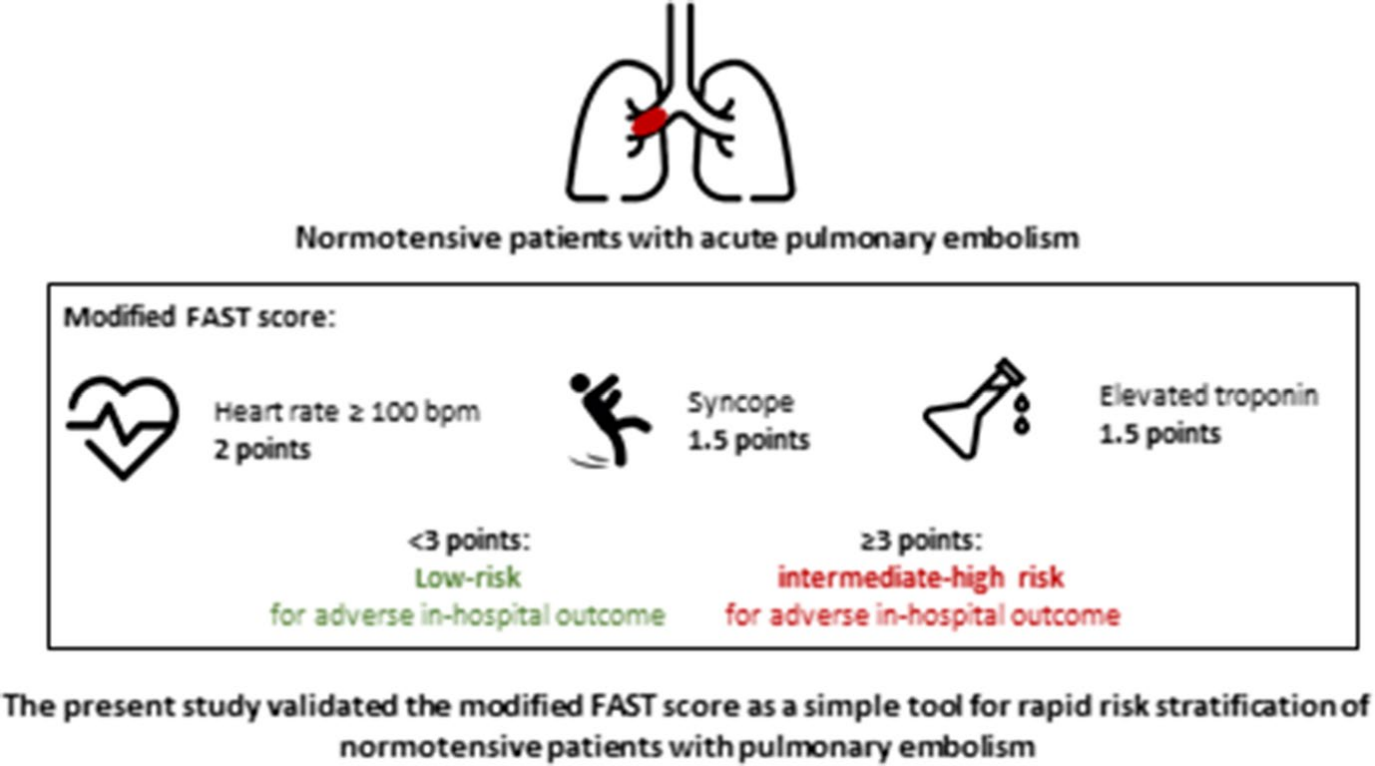

**Electronic supplementary material:**

The online version of this article (10.1007/s00392-019-01593-w) contains supplementary material, which is available to authorized users.

## Introduction

The 2019 guidelines of the European Society of Cardiology (ESC) emphasizes the importance of risk stratification of the heterogeneous group of normotensive patients with pulmonary embolism (PE) to define the appropriate management strategy [1]. While patients with low risk of an early adverse outcome or death may be candidates for early discharge and continuation of treatment at home [2, 3], early reperfusion treatment (e.g. systemic thrombolysis) should be considered for patients with intermediate-high risk PE and in appearance of hemodynamic decompensation [4].

However, the identification of the subgroup of normotensive patients with the highest risk of an adverse early outcome and an acceptable risk-to-benefit ratio justifying thrombolytic therapy still remains challenging. The algorithm for risk stratification proposed by the 2019 ESC guideline is characterized by complexity caused by the calculation of a clinical score (preferably the simplified Pulmonary Embolism Severity Index [sPESI]), laboratory testing and imaging procedures. Combination models such as the FAST [5, 6] and the Bova score [7–9] were developed and validated to identify normotensive patients at highest risk of early PE-related complications (Table 1). Since the FAST score requires measurement of heart-type fatty acid-binding protein (H-FABP), a biomarker of myocardial injury, which is not routinely available in the majority of hospitals, we demonstrated that replacement of H-FABP by high-sensitivity troponin T (hsTnT) for calculation of the modified FAST score provides equivalent prognostic information in normotensive PE patients [10].

**Table 1:** Scores for risk stratification of normotensive pulmonary embolism

Score	Bova score [8, 9]		Modified FAST score [10]	
Items (points)	Elevated cardiac troponin	2	Elevated cardiac troponin	1.5
	RV dysfunction (TTE or CT)	2	Syncope	1.5
	Heart rate ≥ 110 bpm	1	Heart rate ≥ 100 bpm	2
	Systolic blood pressure 90–100 mmHg	2		
*Risk classes*				
Low risk	0–2 points		< 3 points	
Intermediate-low risk	3–4 points		–-	
Intermediate-high risk	> 4 points		≥ 3 points	

*sPESI* simplified Pulmonary Embolism Severity Index, *FAST, H-FABP* syncope, tachycardia, *H-FABP* heart-type fatty acid-binding protein, *RV* right ventricular; *TTE* transthoracic echocardiography, *CT* computed tomography, *bpm* beat per minute, *hsTnT* high-sensitivity troponin T

In the present study we aimed to validate the prognostic performance of the modified FAST score for risk stratification of normotensive PE in a prospective observational multicenter registry and to compare its prognostic performance with established risk prediction models such as the algorithm proposed by the 2019 ESC guideline and the Bova score.

## Material and methods

### Patient cohort and study design

Consecutive normotensive (systolic blood pressure ≥ 90 mmHg on admission) patients aged ≥ 18 years with confirmed acute PE were included in the observational multicentre Italian Pulmonary Embolism Registry (IPER) between January 2006 and November 2010. The study protocol has been described in detail previously [11]. Patients were excluded from the present analysis if they fulfilled at least one of the following criteria: 1) missing troponin plasma concentrations on admission or 2) missing information on right ventricular (RV) on imaging. RV dysfunction was assessed by transthoracic echocardiography (TTE) and defined as (1) right-to-left ventricular/end-diastolic diameter ratio ≥ 1 in apical four-chamber view, (2) right-to-left ventricular/end-diastolic diameter ratio ≥ 0.6 in parasternal long-axis or subcostal four-chamber view and (3) right ventricular-to-right atrial pressure gradient ≥ 30 mmHg [1]. Troponin elevation on admission was defined as troponin I or troponin T plasma concentrations above the assay-specific cut-off value, respectively.

Patients were stratified post-hoc in risk classes according to the sPESI [12], the modified FAST [10] and the Bova score [8] (Table 1). For classification of patients according the algorithms proposed by 2014 and 2019 ESC guidelines [1, 13], patients with a sPESI of 0 points and elevation of cardiac troponin or signs of RV dysfunction on TTE were classified as intermediate-low risk [1, 13]. For calculation of all algorithms and scores, missing values were considered to be normal [14].

All patients were followed for the in-hospital stay. The primary outcome was an in-hospital adverse outcome defined as PE-related death, need for mechanical ventilation, cardiopulmonary resuscitation or administration of catecholamines (except for dopamine at an infusion rate of ≤ 5 µg/kg of body weight per minute). The secondary outcome was in-hospital all-cause mortality.

Study results were not communicated to the clinicians and thus not used to guide the patient management or to monitor the effects of treatment during the hospital stay. The study protocol was conducted in accordance with the amended Declaration of Helsinki and was approved by the local independent Ethic Committees at the study centres. All patients gave informed written consent for participation in the study.

### Statistical analysis

The Fisher´s exact test or Chi^2^ test was used to compare categorical variables, which are expressed as absolute number or percentage. Continuous variables were found not to follow a normal distribution when tested with the modified Kolmogorov–Smirnov test (Lilliefors test); therefore, these variables are expressed as medians with the corresponding interquartile range (IQR) and were compared using the unpaired Mann–Whitney *U* test. Receiver operating characteristics (ROC) curve analysis was performed to determine the area under the curve (AUC) of algorithms and scores (absolute points) with regard to study outcomes. To allow comparison of algorithms and scores, the three-level 2019 ESC algorithm and Bova score were dichotomized as low- and intermediate-low risk (low risk) versus intermediate-high risk (intermediate-high risk). The McNemar–Bowker test was used to compare the distribution of patients by the classification of different dichotomous risk assessment strategies. Comparison of the prognostic performance of dichotomous algorithms and scores was performed by calculation of sensitivity, specificity, positive predictive value (PPV), negative predictive value (NPV), positive and negative likelihood ratios (LR). The prognostic relevance of dichotomous algorithms and scores as well as single predictors with regard to study outcomes was tested using univariate and multivariate (adjusted for age and sex) logistic regression analysis and presented as Odds ratios (OR) with corresponding 95% confidence intervals (CIs). A two-sided significance level of *α* < 0.05 was defined appropriate to indicate statistical significance. Statistical analyses were performed using SPSS (version 21.0, SPSS Inc., Chicago, Illinois, USA).

## Results

### Baseline findings and clinical outcomes

Between January 2006 and November 2010, 1,515 normotensive patients ≥ 18 years with objectively confirmed PE were included in IPER and 647 patients (42.7%) excluded from the present analysis because of missing troponin plasma concentrations and / or missing information on RV dysfunction on TTE (Figure S1 of the supplementary material). The baseline characteristics, medical history and initial presentation of the 868 study patients (57.3%) are shown in Table 2, left column. Troponin I was measured in 701 patients (80.8%) and troponin T in 167 patients (19.2%). Troponin levels were elevated in 426 patients (49.1%) and 621 patients (71.5%) had RV dysfunction on TTE. Overall, 87 patients (10.0%) received reperfusion therapy (85 underwent thrombolysis and 2 percutaneous embolectomy). During the in-hospital stay, 27 patients (3.1%) had an adverse outcome and 32 patients (3.7%) died. Patients died of PE (37.5%), intracranial haemorrhage (12.5%), cancer (9.4%) or other not defined further causes (37.5%).

**Table 2:** Baseline characteristics, medical history and initial presentation of 868 normotensive patients with acute pulmonary embolism

	All study patients (*n* = 868)	Modified FAST score ≥ 3 points (*n* = 302)	Modified FAST score < 3 points (*n* = 566)	*p* value
Sex (male)	389/868 (44.8%)	140/302 (46.4%)	249/566 (44.0%)	0.520
Age (years)	70/868 (63–81)	70/302 (62–81)	70/566 (64–81)	0.930
*Risk factors for VTE and comorbidities*				
Previous VTE	180/868 (20.7%)	57/302 (18.9%)	138/566 (24.4%)	0.073
Cancer*	168/859 (19.6%)	66/301 (21.9%)	102/558 (18.3%)	0.208
Chronic left heart disease	52/867 (6.0%)	14/301 (4.7%)	38/566 (6.7%)	0.293
Coronary artery disease	137/854 (16.0%)	40/295 (13.6%)	97/559 (17.4%)	0.170
*Symptoms and clinical findings on admission*				
Syncope	103/867 (11.9%)	78/302 (25.8%)	25/565 (4.4%)	** < 0.001**
Heart rate (bpm)	98/866 (82–110)	112/301 (101–120)	90/565 (78–100)	** < 0.001**
Heart rate ≥ 100 bpm	441/866 (50.8%)	283/301 (93.7%)	158/565 (27.9%)	** < 0.001**
Heart rate ≥ 110 bpm	270/866 (31.1%)	173/301 (57.3%)	97/565 (17.1%)	** < 0.001**
Systolic blood pressure (mmHg)	131/868 (115–140)	127/302 (110–140)	133/566 (120–140)	** < 0.001**
Mild hypotension†	10/868 (1.2%)	6/302 (2.0%)	4/566 (0.7%)	0.105
Hypoxaemia‡	235/655 (35.9%)	231/235 (98.3%)	401/420 (95.5%)	0.076
RV dysfunction on TTE	621/868 (71.5%)	270/302 (89.4%)	351/566 (62.0%)	** < 0.001**
Elevated troponin	426/868 (49.1%)	284/302 (94.0%)	142/566 (25.1%)	** < 0.001**
*Treatment and in-hospital outcomes*				
Reperfusion therapy	87/867 (10.0%)	52/302 (17.2%)	35/565 (6.2%)	** < 0.001**
Adverse outcome	27/868 (3.1%)	16/302 (5.3%)	11/566 (1.9%)	**0.006**
PE-related death	12/868 (1.4%)	7/302 (2.3%)	5/566 (0.9%)	0.123
All-cause death	32/868 (3.7%)	17/302 (5.6%)	15/566 (2.7%)	**0.036**

^*^ Defined as active or anti-tumor therapy within the last 6-months, or metastatic state

^†^ Defined as systolic blood pressure between 90 and 100 mmHg on admission

^‡^ Arterial oxygen saturation < 90%

*FAST* H-FABP, syncope, tachycardia, *H-FABP* heart-type fatty acid-binding protein, *VTE* venous thromboembolism, *PE* pulmonary embolism, *bpm* beats per minute, *RV* right ventricular, *TTE* transthoracic echocardiography

### Prognostic performance of the modified FAST score

Overall, 302 patients (34.8%) were classified as intermediate-high risk (≥ 3 points) using the modified FAST score. As shown in Table 2, these patients presented more often with symptoms and clinical findings indicating more severe PE and more often reached the primary (in-hospital adverse outcome, 5.3% vs. 1.9%, p = 0.006) and the secondary (all-cause mortality, 5.6% vs. 2.7%, p = 0.036) outcome compared to patients classified as low risk. A modified FAST score ≥ 3 points was associated with a 2.8-fold increased risk (Odds ratio) to reach the primary (95% CI, 1.3–6.2; *p* = 0.009; Tables 3, 4) and a 2.2-fold increased risk to reach the secondary (95% CI, 1.1–4.5; *p* = 0.030) outcome.

**Table 3:** Prognostic performance of risk assessment strategies with regard to in-hospital adverse outcome

	OR (95% CI), *p* value	AUC (95% CI)	Sensitivity (95% CI)	Specificity (95% CI)	PPV (95% CI)	NPV (95% CI)	Negative LR (95% CI)	Positive LR (95% CI)
Modified FAST score	2.8 (1.3–6.2) p = 0.009	0.67 (0.59–0.76)	0.59 (0.41–0.75)	0.66 (0.63–0.69)	0.05 (0.03–0.08)	0.98 (0.97–0.99)	1.7 (1.3–2.4)	0.6 (0.4–0.9)
Bova score*	1.6 (0.7–3.7) p = 0.266	0.64 (0.55–0.73)	0.29 (0.16–0.48)	0.59 (0.54–0.64)	0.04 (0.02–0.08)	0.97 (0.96–0.98)	1.4 (0.8–2.6)	0.9 (0.7–1.1)
2019 ESC algorithm*	4.2 (1.9–9.0) p < 0.001	0.68 (0.57–0.78)	0.52 (0.34–0.70)	0.79 (0.77–0.82)	0.07 (0.05–0.12)	0.98 (0.97–0.99)	2.5 (1.7–3.7)	0.6 (0.4–0.9)

^**^The three-level 2019 ESC algorithm and Bova score were dichotomized as low- and intermediate-low risk (low risk) versus intermediate-high risk (intermediate-high risk)

*ESC* European Society of Cardiology, *FAST* H-FABP, syncope, tachycardia, *H-FABP* heart-type fatty acid-binding protein, *OR* odds ratio, *AUC* area under the curve, *CI* confidence interval, *PPV* positive predictive value, *NPV* negative predictive value, *LR* likelihood ratio

**Table 4:** Prognostic performance of risk assessment strategies with regard to in-hospital all-cause mortality

	OR (95% CI), *p* value	AUC (95% CI)	Sensitivity (95% CI)	Specificity (95% CI)	PPV (95% CI)	NPV (95% CI)	Negative LR (95% CI)	Positive LR (95% CI)
Modified FAST score	2.2 (1.1–4.5) *p* = 0.030	0.65 (0.57–0.72)	0.53 (0.46–0.69)	0.66 (0.63–0.69)	0.06 (0.04–0.09)	0.97 (0.96–0.98)	1.6 (1.1–2.2)	0.7 (0.5–1.0)
Bova score*	1.3 (0.6–2.9) *p* = 0.569	0.62 (0.53–0.70)	0.25 (0.13–0.42)	0.79 (0.76–0.82)	0.04 (0.02–0.08)	0.97 (0.95–0.98)	1.2 (0.7–2.2)	0.9 (0.8–1.16)
2019 ESC algorithm*	5.8 (2.8–12.0) *p* < 0.001	0.73 (0.64–0.81)	0.59 (0.42–0.74)	0.80 (0.77–0.82)	0.10 (0.07–0.15)	0.98 (0.97–0.99)	3.0 (2.2–4.1)	0.5 (0.3–0.8)

^**^The three-level 2019 ESC algorithm and Bova score were dichotomized as low- and intermediate-low risk (low risk) versus intermediate-high risk (intermediate-high risk)

*ESC* European Society of Cardiology, *FAST* H-FABP, syncope, tachycardia, *H-FABP* heart-type fatty acid-binding protein, *OR* odds ratio, *AUC* area under the curve, *CI* confidence interval, *PPV* positive predictive value, *NPV* negative predictive value, *LR* likelihood ratio

### Comparison of algorithms and scores

**Fig. 1:**
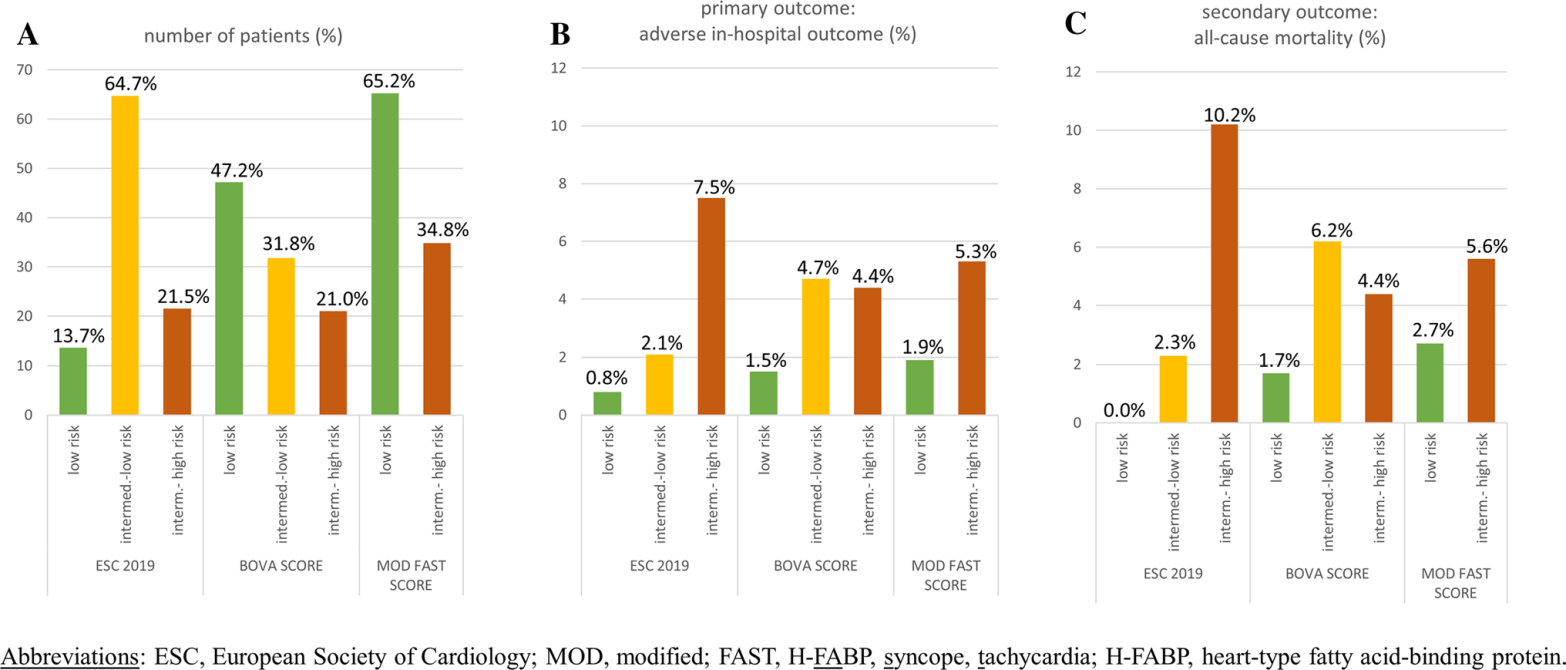
Performance of algorithms and score for risk assessment of acute PE. Classification in risk classes (**a**), rate of an in-hospital adverse outcome (**b**) and in-hospital all-cause death (**c**)

As shown in Fig. 1a, b, the Bova and the modified FAST score classified a larger number of patients in the low risk classes than the 2019 ESC algorithm (*p* < 0.001, McNemar-Bowker test); of those, 1.5% and 1.9%, respectively, had an in-hospital adverse outcome. Only one patient (0.8%) had an in-hospital adverse outcome if stratified as low risk by the 2019 ESC algorithm. The modified FAST score classified more patients (34.8%) as intermediate-high risk than the Bova score (21.0%). However, the rate of an in-hospital adverse outcome was numerically higher for patients classified as intermediate-high risk by the modified FAST score compared to the Bova score (5.3% vs. 4.4%) and highest for patients classified as intermediate-high risk by the 2019 ESC algorithm (7.5%). Except for the Bova score, the rate of an in-hospital adverse outcome was highest in the intermediate-high risk classes while all patients classified in low risk classes had a PE-related mortality rate of less than 2% (Fig. 1b).

**Fig. 2:**
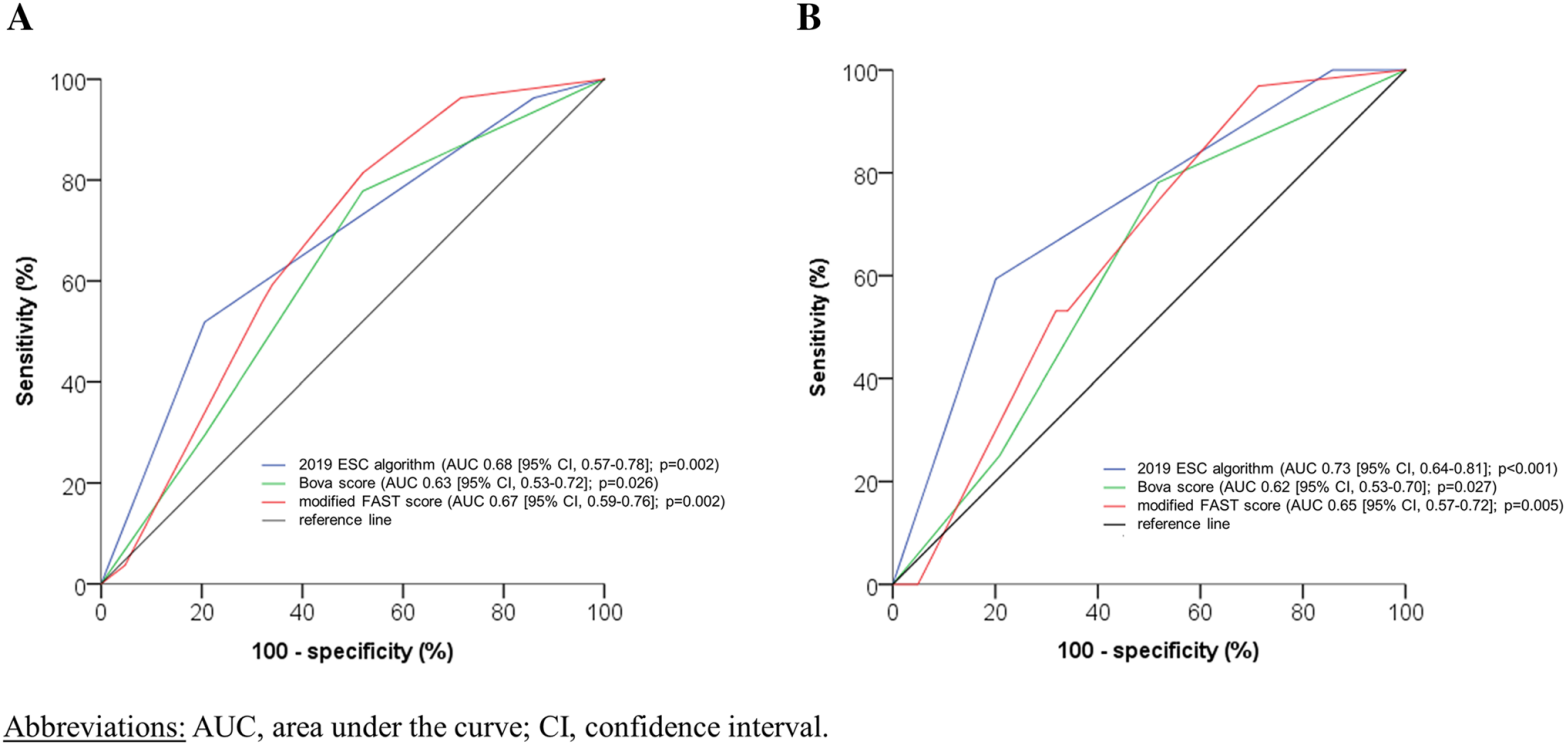
Receiver operating characteristics (ROC) analysis of risk assessment strategies with regard to an in-hospital adverse outcome (**a**) and all-cause mortality (**b**)

ROC analyses revealed a larger AUC with regard to an in-hospital adverse outcome for the modified FAST score (AUC, 0.67; 95% CI, 0.59–0.76; *p* = 0.002) compared to the Bova score (AUC, 0.64; 95% CI, 0.56–0.73; *p* = 0.012) (Table 3a; Fig. 2a). A larger OR with regard to an in-hospital adverse outcome was observed for patients classified as intermediate-high risk by the 2019 ESC algorithm (OR, 4.2; 95% CI 1.9–9.0; *p* < 0.001) and the modified FAST score (OR, 2.8; 95% CI, 1.3–6.2; *p* = 0.009) compared to the Bova score, which failed to identify patients at higher risk (OR, 1.6; 95% CI, 0.7–3.7; *p* = 0.266; Table 3).

While the 2019 ESC algorithm (OR, 5.8; 95% CI, 2.8–12.0; *p* < 0.001) and the modified FAST score (OR, 2.2; 95% CI, 1.1–4.5; *p* = 0.030) were able to predict in-hospital all-cause mortality, the Bova score (OR, 1.3; 95 CI, 0.6–2.9, *p* = 0.569) did not provide prognostic information with regard to the secondary outcome. Overall, the ESC 2019 algorithm provided the best risk prediction for all-cause mortality with a sensitivity of 59% and a positive predictive value (PPV) of 10% (Table 4).

In addition, we tested the prognostic performance of comorbidities and clinical findings on admission with regard to the prediction of an in-hospital adverse outcome. Multivariate logistic regression analysis indicated an independent (adjusted for age and sex) prognostic value of active cancer, tachycardia and RV dysfunction on TTE with regard to an in-hospital adverse outcome (Table 5).

**Table 5:** Predictors of an in-hospital adverse outcome

	OR (95% CI)	*p* value	OR (95% CI)	*p* value
	Univariate		Multivariate (adjusted for age and sex)	
*Comorbidities*				
Cancer	2.5 (1.1–5.6)	0.024	2.7 (1.2–6.3)	0.015
Chronic left heart disease	1.2 (0.3–5.5)	0.754	1.0 (0.4–2.7)	0.931
*Symptoms and clinical findings on admission*				
Syncope	1.3 (0.4–3.8)	0.633	1.3 (0.4–3.7)	0.668
Heart rate ≥ 100 bpm	3.5 (1.4–8.8)	0.007	3.8 (1.5–9.5)	0.005
Heart rate ≥ 110 bpm	1.5 (0.7–3.4)	0.275	1.6 (0.7–3.6)	0.215
RV dysfunction on TTE	5.1 (1.2–21.9)	0.027	4.8 (1.1–20.3)	0.036
Elevated troponin	2.1 (0.9–4.8)	0.024	2.0 (0.9–4.5)	0.108

Definitions are provided in the footnote of Table 2

*OR* odds ratio, *CI* confidence interval, *bpm* beats per minute, *RV* right ventricular, *TTE* transthoracic echocardiography

## Discussion

We performed a post-hoc analysis of a large Italian multicentre registry to validate the prognostic impact of the modified FAST score in 868 normotensive patients with acute PE. The main study findings can be summarized as follows: (1) the modified FAST score allows accurate risk assessment and predicts PE-related complications and all-cause mortality, (2) the Bova score was not able to discriminate between patients with intermediate-low and intermediate-high risk of adverse outcomes and (3) the 2019 ESC algorithm showed the best prognostic performance for the identification of normotensive PE patients at highest risk of adverse outcomes.

The 2014 and the recently presented 2019 ESC guidelines suggest that the assessment of clinical variables, myocardial injury markers and RV function on imaging allows the identification of normotensive PE patients at higher risk who require monitoring and reperfusion therapy if haemodynamic decompensation appears [1, 13]. Several cohort studies demonstrated that the 2014 ESC algorithm reliably stratifies PE patients in different risk categories [10, 15–18]. Furthermore, a recently published meta-analysis including 7,536 patients from 22 studies confirmed the importance of RV dysfunction, even in “low risk” patients identified by clinical criteria (PESI < 86 points, sPESI 0 points or all Hestia criteria absent) [3]. Overall, 34% (95% CI, 30–39%) of these “low-risk” patients had signs of RV dysfunction on TTE or computed tomography (CT) which was associated with a higher rate of an early adverse outcome (PE-related mortality, haemodynamic collapse or recurrent VTE) compared to patients with absence of RV dysfunction (3.7%; 95% CI, 0.9–14.4% versus 0.7%; 95% CI, 0.06–6.40%). Concordantly, in the present study, the rate of an in-hospital adverse outcome was considerable high (1.5%) if classification to the low risk group was based on a negative sPESI only (data not shown). The lower (0.8%) mortality rate observed in patients with a negative sPESI *and* absence of RV dysfunction and troponin elevation indicates that imaging and laboratory biomarker testing should be performed before treatment decisions such as early discharge are made. This concept has recently been tested in a management trial confirming that early discharge and home treatment with rivaroxaban is effective and safe in carefully selected patients with acute low-risk PE (based on the modified Hestia criteria and absence of RV dysfunction on imaging) [2]. On the other hand, normotensive PE patients with RV dysfunction have a high mortality rate of up to 16.4% [19] and, in the present study, an 4.8-fold increased risk for an in-hospital adverse outcome independently of age and sex. Besides the prognostic importance of RV dysfunction, the present study underlines the relevance of comorbidities by identifying cancer as an important independent predictor of an in-hospital adverse outcome. This is further indicated by a higher sensitivity and positive predictive value of the 2019 ESC algorithm (including cancer and other comorbidities for calculation of the sPESI) in comparison to other risk assessment strategies not considering comorbidities.

However, given the complexity caused by the need for calculation of the sPESI, laboratory testing and imaging for stratifying patients to risk classes using the 2019 ESC guidelines algorithm, in the past years, a number of clinical prediction scores were developed aiming to optimize and simplify risk assessment. The modified FAST score is easy and fast to calculate and only requires information on troponin levels, tachycardia and syncope. The fact that TTE is not needed for calculation of the modified FAST scores constitutes an advantage considering the poor standardization of criteria for the definition of RV dysfunction on TTE and the lack of availability of TTE outside the working hours in many hospitals [20, 21]. Further, syncope is included as a clinical variable in the FAST score and was identified in the derivation and validation study as a predictor of an adverse early outcome (OR, 5.05 [95% CI, 1.42–17.94] and OR, 5.11 [95% CI, 1.76–14.83], respectively) [5, 6]. In patients with acute PE, syncope is considered to result from the sudden obstruction of pulmonary arteries due to embolized thrombi leading to a transient drop of cardiac output. A recent meta-analysis including 21,956 patients from 29 studies demonstrated that syncope is associated with a higher prevalence of haemodynamic instability in PE patients [22]. In contrast to these findings, in the present study, syncope was not identified as a predictor of adverse outcome. However, given the lack of a standardised definition of syncope – which was the case both in the present study and in the earlier studies included in the meta-analysis – the clinical relevance of these observations remains unclear and apparent discrepancies should not be overinterpreted.

Since the FAST score requires measurement of H-FABP, a biomarker of myocardial injury, which is not routinely available in the majority of hospitals, replacement with another myocardial injury marker, namely troponin, seems plausible. The prognostic performance of elevated troponin levels has been confirmed by numerous studies and troponin elevation was used as an inclusion criteria in a randomized trial investigating thrombolysis in normotensive PE patients [4]. A meta-analysis including 1,985 patients from 20 studies demonstrated that elevated troponin levels were associated with a 5.9-fold (95% CI, 2.68–12.95) increased risk for mortality in normotensive PE patients [23]. We demonstrated in 388 normotensive PE patients from a single-centre cohort study that a modified FAST score (with replacement of H-FABP by hsTnT) provides equivalent prognostic information and a modified FAST score ≥ 3 points was associated with a 16-fold (95% CI, 5.3–47.6; *p* < 0.001) increased risk for PE-related complications [10]. In the present study, we were able to validate our previous findings and demonstrate that a positive modified FAST score (≥ 3 points or 2 items present) is associated with a 2.8-fold (95% CI, 1.3–6.2; *p* = 0.009) increased risk for an in-hospital adverse outcome. Interestingly, every sixth patients classified as intermediate-high risk by the modified FAST score received systemic thrombolysis. However, a modified FAST score ≥ 3 points was associated with a PPV of 5% only; thus, further studies are needed before recommendations for more aggressive treatment (such as thrombolysis) of patients classified as high risk based on the modified FAST score can be made. Further, as the (modified) FAST score was developed to identify normotensive patients with a high risk of PE-related complications, a small but relevant number of patients with a (modified) FAST score of < 3 points had an adverse outcome, both in previous studies [5, 6, 10] and in the present one. Thus, the (modified) FAST score should not be used to identify candidates for home treatment.

Besides the modified FAST score, the Bova score was developed to identify patients at higher risk for adverse early events. Patients classified in class III (> 4 points, intermediate-high risk) had a rate of an adverse outcome of 29.2% in the derivation and of 42.0% and 7.5% in validation studies, respectively [7–9]. However, in the present study, the Bova score was not able to discriminate between patients at intermediate-low (4.7% in-hospital adverse outcome rate) and intermediate-high risk (4.4% in-hospital adverse outcome rate) and was associated with a lower sensitivity (29%) and a lower positive predictive value (4%) compared to the 2019 ESC algorithm (52% and 7%, respectively) and the modified FAST score (59% and 5%, respectively).

Some potential limitations of our study may deserve consideration: First, the rate of patients with PE-related death (1.4%) was lower compared to other cohort studies and the derivation study limiting the power of statistical analyses [8, 10]. Second, only patients with RV assessment on TTE and troponin measurements were included in the study; thus, the number of low risk patients might have been underestimated. Moreover, data on RV dysfunction on CT or other biomarkers such as natriuretic peptides, lactate or copeptin were not available. Third, for calculation of the sPESI, missing variables were considered to be normal. However, the strength of our study is the multicentre prospective design and the large sample size.

In conclusion, in the present study we were able to validate the modified FAST score as a simple tool for rapid risk stratification of patients with acute PE, while the Bova score failed to discriminate between intermediate-low and intermediate-high risk patients. As the 2019 ESC algorithm provided the best prognostic performance, it should be used for risk assessment in clinical routine. In this regard, our findings underline the importance of assessing signs of RV dysfunction on imaging, even in “low-risk” PE patients. However, if TTE is not immediately available, the modified FAST score may serve as a fast and simple tool to identify normotensive patients at high risk of PE-related complications.

## Electronic supplementary material

Below is the link to the electronic supplementary material.

Supplementary material 1 (TIF 937 kb)

Supplementary material 2 (TIF 728 kb)

Supplementary material 3 (TIF 651 kb)

## Abbreviations

AUC: Area under the curve
CT: Computed tomography
CI: Confidence interval
ESC: European Society of Cardiology
FAST score: H-FABP, syncope and tachycardia
GFR: Glomerular filtration rate
H-FABP: Heart-type fatty acid-binding protein
IPER: Italian Pulmonary Embolism Registry
hsTnT: High-sensitivity troponin T
IQR: Interquartile range
LR: Likelihood ratio
LV: Left ventricular
LWMH: Low-weight molecular heparin
NT-proBNP: N-terminal pro-brain natriuretic peptide
NPV: Negative predictive value
OR: Odds ratio
PE: Pulmonary embolism
PPV: Positive predictive value
RV: Right ventricular
ROC: Receiver operating characteristics
(s)PESI: (Simplified) Pulmonary Embolism Severity Index
TTE: Transthoracic echocardiography
VTE: Venous thromboembolism
