# Supplementary figures and images for: Validation of a fast prognostic score for risk stratification of normotensive patients with acute pulmonary embolism
Source: Clin Res Cardiol. 2020 Feb 6;109(8):1008–17. doi: 10.1007/s00392-019-01593-w (PMC7376081; doi:10.1007/s00392-019-01593-w)

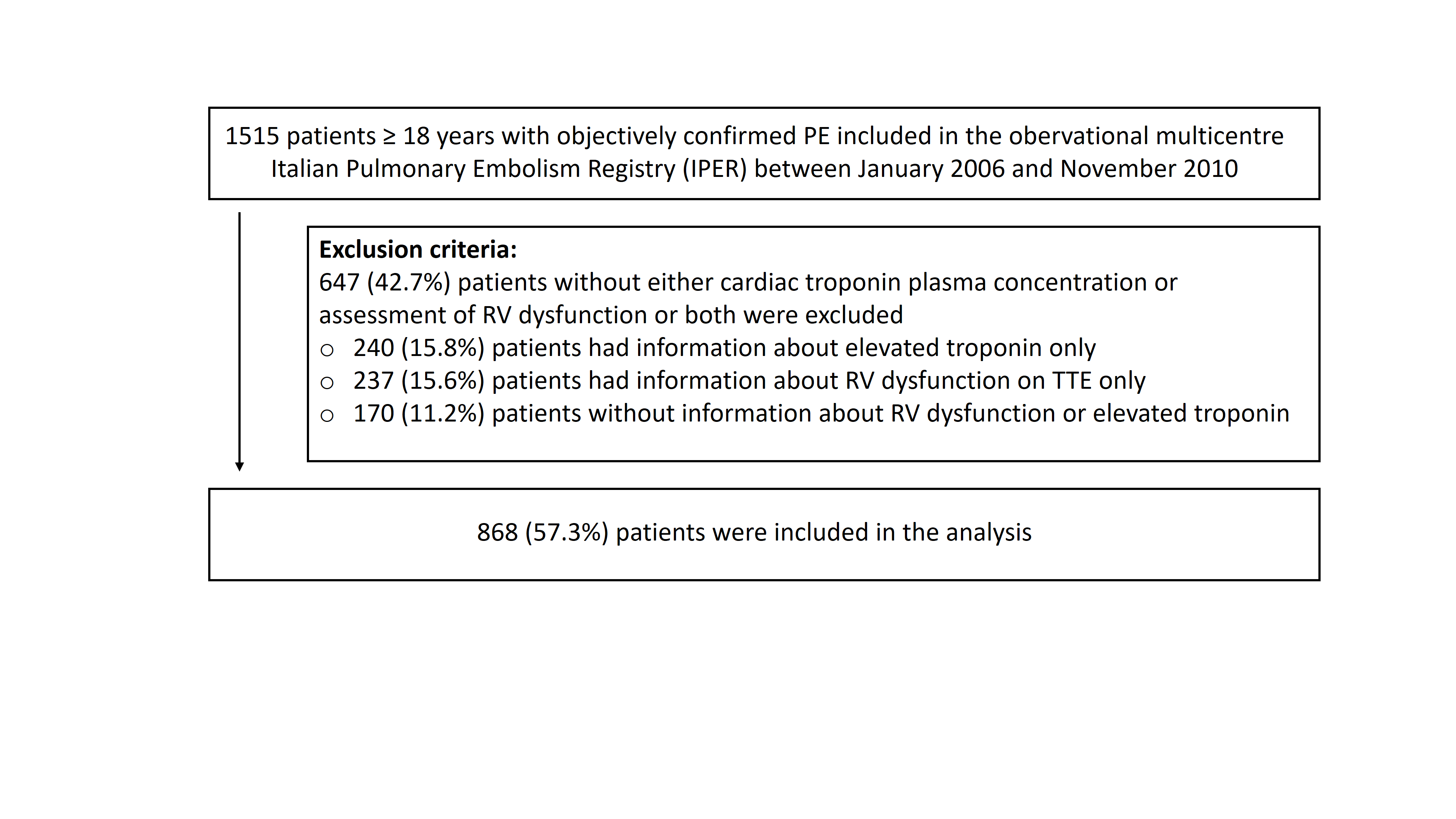

Supplement: Supplementary file 1 — Supplementary material 1 (TIF 937 kb) [file 392_2019_1593_MOESM1_ESM.tif]

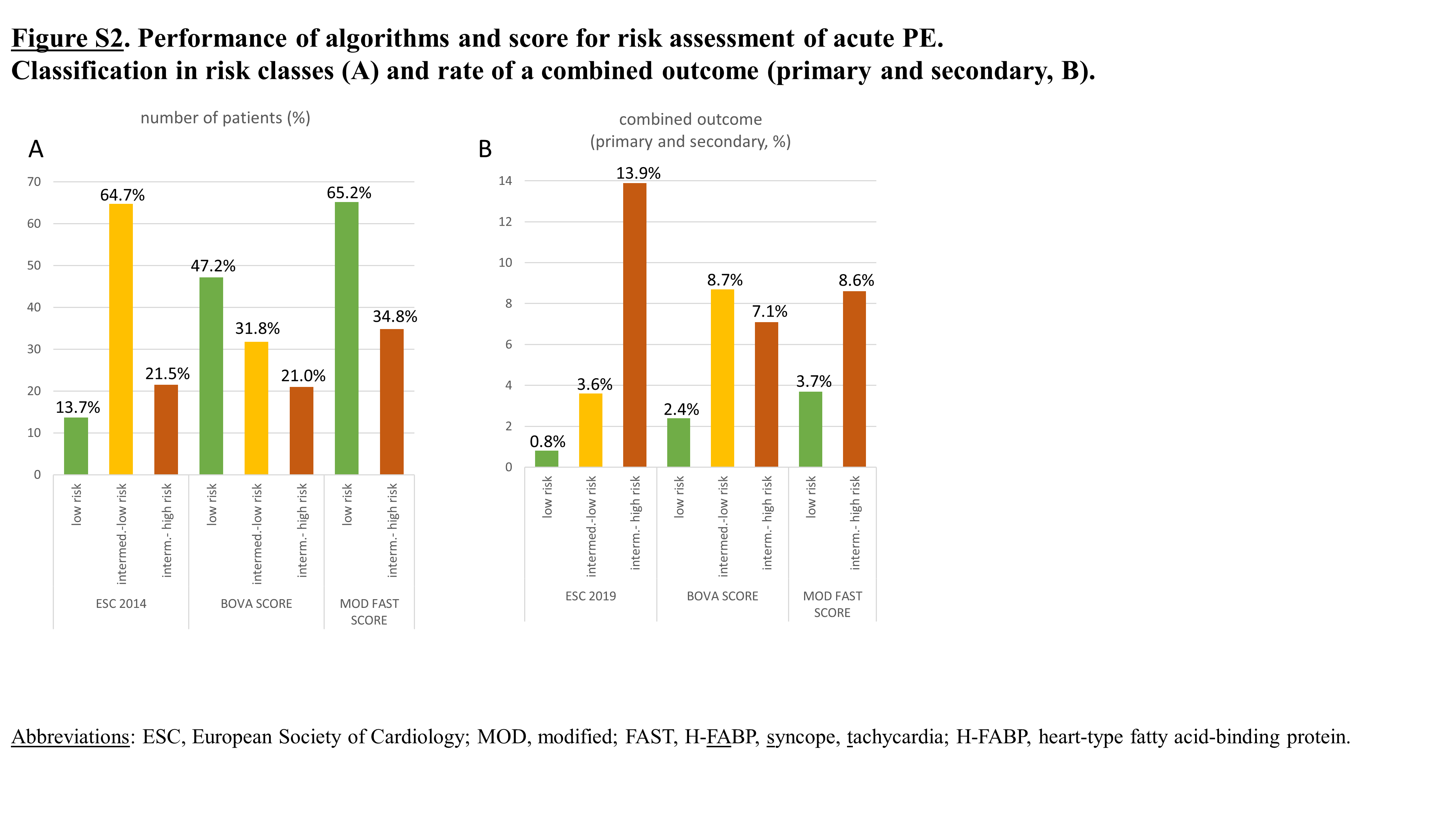

Supplement: Supplementary file 2 — Supplementary material 2 (TIF 728 kb) [file 392_2019_1593_MOESM2_ESM.tif]

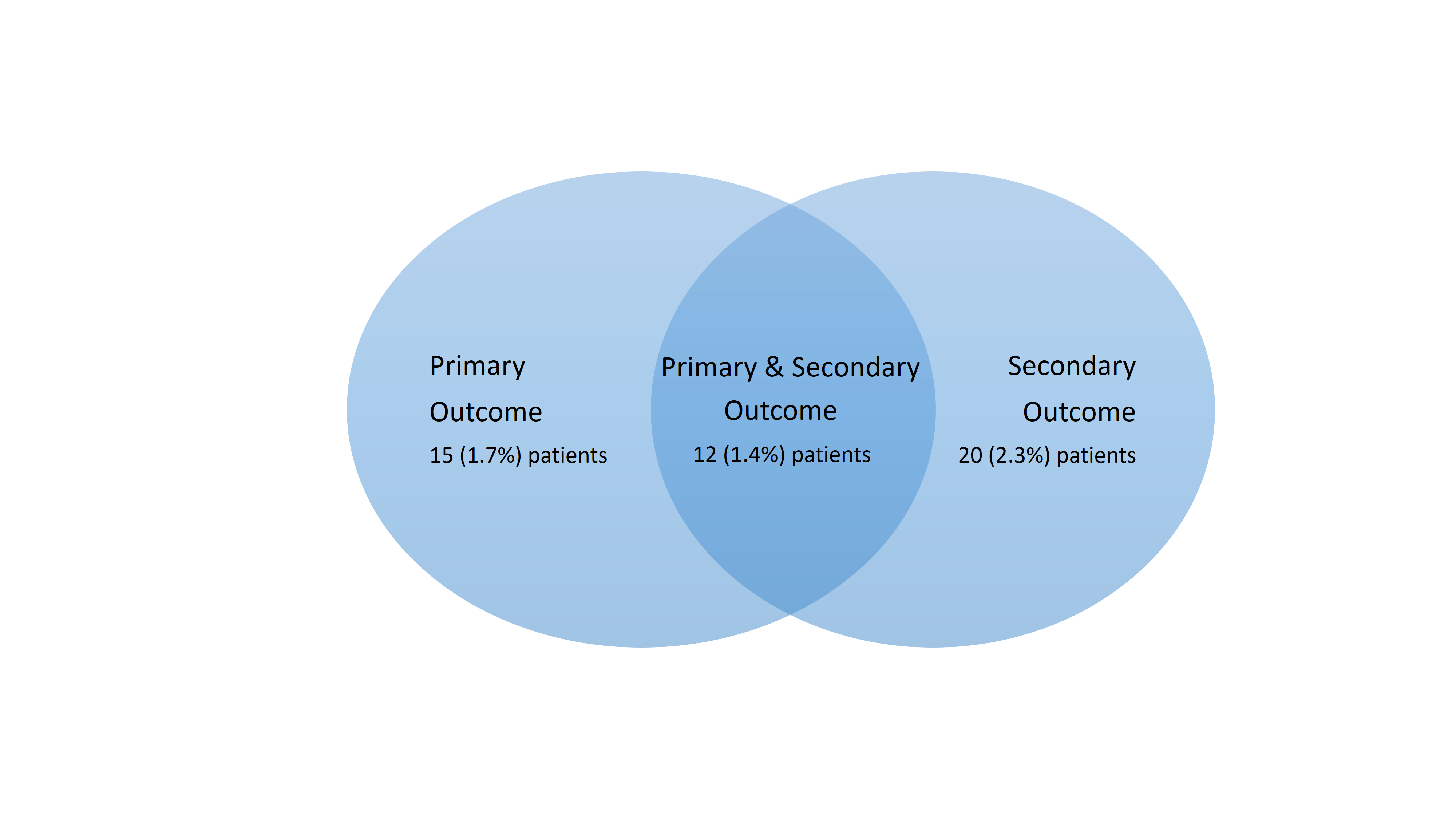

Supplement: Supplementary file 3 — Supplementary material 3 (TIF 651 kb) [file 392_2019_1593_MOESM3_ESM.tif]
